# Nævus Maternus

**Published:** 1828-05

**Authors:** 


### NÆVUS MATERNUS.

### *Case of Nævus Maternus, cured by Vaccination, at the* Royal Infirmary, Glasgow.

Cameron

Jane M'Gibbon, æt. three months, had, on admission, a roundish tumor, nearly as large as a sixpence, on the right side of the chin. It was considerably elevated above the surrounding skin, had a purplish colour, and appeared to be formed of blood-vessels. At birth, the tumor was only the size of a split pea.

Around the border of the tumor, as well as all over its surface, minute punctures were made, and vaccine lymph freely applied. In ten days thereafter, the whole disease was involved in one pustule; but, when this became incrusted and was thrown off, there still remained a dark-coloured, prominent, and diseased surface. Another suppuration succeeded, and a third; when, at the end of five weeks, a complete cure was effected, no trace of the disease remaining, nor mark, further than what follows vaccination on a healthy part.

This cure was hardly completed, when another little child was presented in private practice, with a nævus advancing rapidly, and occupying the middle and edge of the upper eyelid. The same process was followed, with very similar results. A cure was effected, but after a very tedious festering and ulceration. Indeed, it appears evident that nothing further is required to effect a cure than to institute a process of suppuration, by cow-pox, by caustic, or by any thing else. As the most gentle of all these means, vaccination is entitled to a preference. But, when this has been used in the common way, and for the ordinary purpose, and before a mark of this kind has excited much attention, I am inclined to think that an antimonial plaster would constitute by far the best substitute. I am sorry that no case has as yet occurred to give me an opportunity of making the experiment.

Before these two cases fell under my care, a child was admitted into the Infirmary with a nævus about the size of a large almond, occupying the red part of the under lip. It was clipped away, not by triangular excision, but along the line of the lip. Three vessels required ligature, but in ten days the part was covered by healthy cuticle, and no disfiguration occasioned. I am satisfied that this mode should more frequently be employed in extirpating cancerous lips. I have frequently performed it successfully in extensively diseased cases. It is uniformly practised by Baron Dupuytren.—Dr. Young, *in Glasgow Journal.*

